# Exercise‐induced release of troponin

**DOI:** 10.1002/clc.23337

**Published:** 2020-01-24

**Authors:** George A. Stavroulakis, Keith P. George

**Affiliations:** ^1^ Division of Cardiology Igias Melathron Athens Greece; ^2^ Research Institute for Sport and Exercise Sciences Liverpool John Moores University Liverpool UK

**Keywords:** exercise‐induced troponin, troponin release

## Abstract

It is well established that regular physical activity reduces cardiovascular disease risk; however, numerous studies have demonstrated postexercise elevations in cardiac troponin (cTn), indicative of cardiac injury in apparently healthy individuals. The prevalence of these findings in different exercise settings and population groups, as well as potential underlying mechanisms and clinical significance of exercise‐induced cTn release are not yet quite determined. The present review will discuss the cTn response to exercise in light of developing cTn assays and the correlation between postexercise cTn release and cardiac function. Additionally, recent data regarding the potential link between strenuous endurance exercise and its relationship with unfavorable cardiac effects in athletes, as well as the management of patients presenting at emergency care after sport events will be briefly reviewed.

**Abbreviations**
ACS: acute coronary syndrome
CAD: coronary artery disease
CK‐MB: creatine kinase‐myocardial band
cMRI: cardiovascular magnetic resonance
CTCA: computed tomography coronary angiography
cTn: cardiac troponin
cTnI: cardiac troponin I
cTnT: cardiac troponin T
LV: left ventricular
MI: myocardial infarction
RV: right ventricular
Tn: troponin

## 1 INTRODUCTION

Studies carried out since 1987 using early experimental troponin (Tn) assays have shown that prolonged exercise may induce detectable increase in circulating troponins.^1^ Driven by the development of more sensitive and specific assays, cardiac Tn (cTn) have progressively formed the cornerstone for the diagnosis of acute myocardial infarction (MI), beginning from the first definition^2^ in 2000, until the recently published Fourth universal definition of MI^3^ in 2018. It is recognized that many nonacute coronary syndrome (ACS) diagnoses and other conditions may also promote cTn elevation, including prolonged exercise.^4^

The introduction of the high‐sensitivity troponin assays, unsurpassed in sensitivity to detect low levels of myocardial damage (possibly at the expense of decreased specificity)^5^ has allowed better characterization of exercise‐induced cTn elevation. This has led to the observation that measurable changes in cTn are common, not only with extreme levels of exercise, such as marathon,^6^ but even following normal physical activity,^7^ or a treadmill test.^8^ The origin of this biomarker release and whether it reflects a physiological or pathological process, remains a contentious issue.^9^ The clinical implications are worthy of study for a number of reasons. First, this issue may be of relevance to the controversy surrounding the long‐term prognosis of athletes presenting with postexercise cTn elevations and the possible role of high level exercise as a cause of cardiac disease in some individuals.^10^ Although the beneficial effect of moderate duration exercise on cardiovascular health in the general population is well recognized,^11^ it has been postulated that participation in multiple extreme endurance events may lead to right ventricular (RV) dysfunction,^12^ accelerate coronary atherosclerosis,^13^ or even promote cardiac fibrosis.^14^ Second, exercise‐induced cTn elevation is important to understand in the management of athletes presenting with chest pain at emergency care after sport events, since in many relevant studies cTn levels exceeded the 99 percentile of the method formally fulfilling the criteria for acute MI.^15^

Previous reports have repeatedly confirmed that cTnT levels at rest can be predictive of future cardiovascular events, not only in the context of CAD or other diseases,^16^, ^17^ but even in apparently healthy individuals.^18^ In contrast, cTn elevation postexercise has been commonly considered a physiological response and a benign, physiological phenomenon in terms of prognosis^19^, ^20^; nevertheless, this theory has been challenged by recent studies suggesting that exercise‐induced cTn release may be related to an increased incidence of adverse cardiovascular events,^21^ or occult obstructive CAD.^22^

This review will summarize available data with regards to the cTn response to exercise and will evaluate this data with reflection on research design, exercise stimulus, participant selection as well as assay development and cTn biochemistry. Potential mechanism, clinical significance, and the management of patients with cTn elevation after exercise will be briefly discussed.

## 2 METHODS

We performed a review of studies related to the cTn response to exercise. A review of research published in English until September 2019 was performed, by conducting systematic searches of PubMed, Scopus and the Cochrane Library, using the Key words “Exercise‐induced troponin,” “Troponin release,” “Troponin physiology,” “Cardiac biomarkers and exercise,” and “Exercise‐induced cardiac damage,” “Exercise‐induced cardiac injury.” Studies that were repetitions were removed. Additional studies were excluded by reading the headlines or abstracts, if they did not concern exercise‐induced elevation of troponin in healthy subjects, or if there were not available in English. The reference lists of the retrieved articles and the review articles published on the subject were also screened for eligible manuscripts. All studies were published between 1987 and 2019.

## 3 RESULTS AND DISCUSSION

### 3.1 Exercise factors

Early reports of exercise‐induced elevations in serum biomarkers, mainly myocardial band isoform of creatine kinase (CK‐MB), led to the concern that high intensity physical activity may result in cardiac injury, which was mitigated by the discovery that CK‐MB lacks specificity regarding cardiac or skeletal muscle origin.^23^ The development of Tn assays has expanded the ability to explore exercise‐induced cardiac injury. Fortescue et al,^6^ in one of the largest relevant reports, studied 482 Boston marathon finishers and found that 68% had some degree of postrace cTn elevation, while 11% of those had increases diagnostic for MI. Interestingly, this increase was found to be more pronounced in less trained athletes, which was confirmed by other reports,^24^ while several others found no association between training status and biomarker release.^25^ Multiple other studies have reported cTn elevations after marathons,^26^ ultradistance races,^27^ triathlon events,^28^ cycling,^29^ and various other forms of physical activity. Exercise intensity and duration cannot reliably predict the magnitude of cTn release.^30^, ^31^, ^32^ Furthermore, aside from extreme sports, detectable biomarker alterations have been described following stress tests,^33^ table tennis games,^34^ or prolonged walking.^35^ It should also be noted that numerous studies found no significant release of cTn, even after high‐intensity exercise.^36^ The discrepancy in reports investigating prevalence and factors affecting the release of cTn directly challenges clinical relevance and supports the hypothesis that it may not actually reflect cardiac injury.

In order to explore a potential association between cTn release and cardiac function, numerous studies have coupled cTn with imaging modalities (echocardiography, cMRI) and biochemical markers (mainly N‐terminal pro‐B‐type natriuretic peptide) after endurance events; nevertheless, results from such efforts have also been inconsistent. (Table 1).

**Table 1:** Studies investigating correlations between cTn elevation and cardiovascular effects

Author	Publication year	Type of exercise	Functional indices	cTn	NT‐proBNP	Correlations between cTn and functional indices
Weippertet al^78^	2016	60 min moderate intensity CT vs sprints with set RPs in‐between	TTE: ‐	↑ in RP		−
O'Hanlon et al^84^	2010	Marathon	CMR: ‐	↑cTnI		−
Neilan et al^39^	2006	Marathon	TTE: diastolic‐RV dysfunction	↑cTnT	↑	+
Mousavi et al^40^	2009	Marathon	TTE, CMR: RV dysfunction	↑cTnT		+
Shave et al^85^	2002	Mountain marathon	TTE: LV dysfunction	↑cTnT		−
George et al^86^	2009	Ultra marathon, 89 km	TTE: ↓ EF, ↓peak strain‐peak systolic, diastolic strain rates	↑cTnT		−
Tulloh et al^87^	2006	Ironman Triathlon	TTE: transient ↓ EF	↑cTnT		+
Rifai et al^88^	1999	Ironman Triathlon	TTE: transient ↓EF	↑cTnT, ±↑cTnI		+ (significant in those with both ↑cTnT and ↑cTnI)
Wilson et al^37^	2011	Marathon	TTE: transient LV ↓ diastolic function CMR: ‐	↑cTnI	↑	−
Whyte et al^89^	2005	Marathon	TTE: LV ↓ diastolic function	↑cTnT		−
George et al^41^	2004	Marathon	TTE: LV ↓ diastolic function	↑cTnT		−
Shave et al^90^	2004	Two 50 mile cycle trials	TTE: ‐	−		−
Scharhag et al^91^	2006	1‐h and a 3‐h exercise study	TTE: ‐ CMR: ‐	↑cTnI	↑	−
La Gerche et al^12^	2012	Endurance race (3‐11 h duration)	TTE: RV dysfunction CMR: RV dysfunction	↑cTnI	↑	Correlations of biomarkers with RVEF but not LVEF
Bohm et al^92^	2016	Cardiopulmonary exercise testing	TTE: ‐ CMR: ‐	↑cTnT		−

*Note*: ↑ means elevation, − means no significant effect, and + means positive correlation.

Abbreviations: cMRI, cardiovascular magnetic resonance; CT, continuous training; EF, ejection fraction; LV, left ventricular; NT‐proBNP, N‐terminal pro‐B‐type natriuretic peptide; RV, right ventricular; TTE, transthoracic echocardiogram.

La Gerche et al^12^ studied 40 athletes following an ultra‐endurance race event and showed that cTnI and B‐type natriuretic peptide were increased postrace and correlated with decrements in RV, but not LV function, as estimated by echo and cMRI, yet, these effects were reversible after a week. In contrast, Wilson et al^37^ found no association between post‐marathon cTnI release and echo/cMRI cardiac function indices. The term “cardiac fatigue” has been used to describe the postexercise decline in systolic and diastolic LV and RV function,^38^ that in most studies do not seem to correlate with cTn elevation. In general, most of the reports incorporating imaging and additional biochemical markers suggest that, even if there is evidence for transient LV or RV dysfunction, there is no solid data suggesting that this is translated to permanent damage.^12^, ^35^, ^39^, ^40^, ^41^ This is in line with good prognosis and longevity of athletes,^42^ even when volume of exercise exceeds significantly the officially recommended 150 minutes per week of moderate intensity or 75 minutes per week of vigorous‐intensity aerobic exercise per week.^43^

### 3.2 STUDY DESIGN FACTORS

The marked heterogeneity in studies regarding postexercise cTn release has been attributed to various factors, including small sample sizes, differences in fitness of participants, varying types and intensity of exercise, different cTn assays, as well as different detection limits used to define a “positive” cTn.^44^ Timing of blood sampling may be a major determinant of this variability,^26^ considering, not only the diversity in cTn elevation kinetics pattern between the well‐studied CAD syndromes vs exercise, but also the uncertainty regarding the actual peak value of cTn. Especially in the context of long duration sport events, multiple or even delayed (24 hours postexercise) sampling may be required in order to disclose a comprehensive trend of cTn.^45^ Furthermore, Kleiven et al,^22^ in a study performed in 120 middle aged predominately male cyclists, showed that cTn elevation 24 hours poststrenuous exercise was more pronounced in subjects with occult obstructive CAD compared to those without significant stenosis, while no difference was observed prior to or 3 hours after the race. Thus, if confirmed by other studies, this indicates that delayed sampling may entail clinical relevance, potentially contributing in the identification of high‐risk individuals.

Meta‐analyses and systematic reviews, that would hopefully yield the adequate overall sample size necessary to provide greater statistical reliability and insights to mechanisms and sources of discrepancy have also been conducted (Table 2). Shave et al,^46^ retrieving data from studies up until 2006, showed that 47% of endurance athletes experienced a significant exercise‐induced rise in cTnT and that 36% of London marathon finishers demonstrated cTnT elevations fulfilling criteria for MI. These results are similar to those reported by others,^47^, ^48^ while the increased prevalence reported in the study be Vilela et al^49^ (70% out of 424 participants retrieved from eight studies showed post‐running cTn values higher than the 99th URL) probably reflects the currently broader utilization of high‐sensitivity assays. Gresslien et al^50^ provide a detailed selection of the more recent relevant studies, but no conclusive data are reported.

**Table 2:** Systematic reviews—meta‐analyses investigating exercise‐induced cTn release

Author	Publication year	Number of studies	Patients included	CTnT (% of subjects with elevation)	Remarks
Shave et al^46^	2007	26	1120	47	Postexercise cTnT after cycling approximately half that of running events (27% vs 52%) Postexercise cTnT not affected by age
Sedaghat‐Hamedani et al^47^	2015	33	1045	51	Average cTnI elevation 40 ng/L c from baseline, no % reported due to significant heterogeneity between studies
Regwan et al^48^	2010	16	939	51	Marathon participants cTn elevation not associated with age and gender, but with publication date and assay sensitivity cTnI less commonly elevated vs cTnT
Vilela et al^49^	2014	10	424	69.8	
Gresslien et al^50^	2016	145		0%‐100%	Extremely thorough citation of studies, but no cumulative data provided

## 4 POTENTIAL MECHANISMS OF EXERCISE‐INDUCED CTN RELEASE

### 4.1 Troponin physiology

The cTn complex, consisting of three proteins known as cTnC, cTnI, and cTnT, forms the skeleton of the striated muscle and regulates actin and myosin cross‐bridge cycling. cTn is bound to tropomyosin on the thin filament of the myofibril, while an undefined proportion remains unbound in cytosolic pool, possibly serving as a reservoir for repair/regeneration of tropomyosin‐bound cTn.^51^ Although skeletal and cTn proteins demonstrate a significant degree of amino acid sequence homology, more than 100 differences exist and they are encoded by separate genes.^52^ Important biological and analytical differences also exist between cTnT and cTnI. During fetal development, cTnT exists in both cardiac and skeletal muscles, but disappears gradually from adult skeletal muscle, yet, it may be reexpressed in case of skeletal muscle injury.^52^, ^53^ TnI has also been previously reported to possess skeletal expression; however, relevant data are scarce.^54^

**Figure 1:**
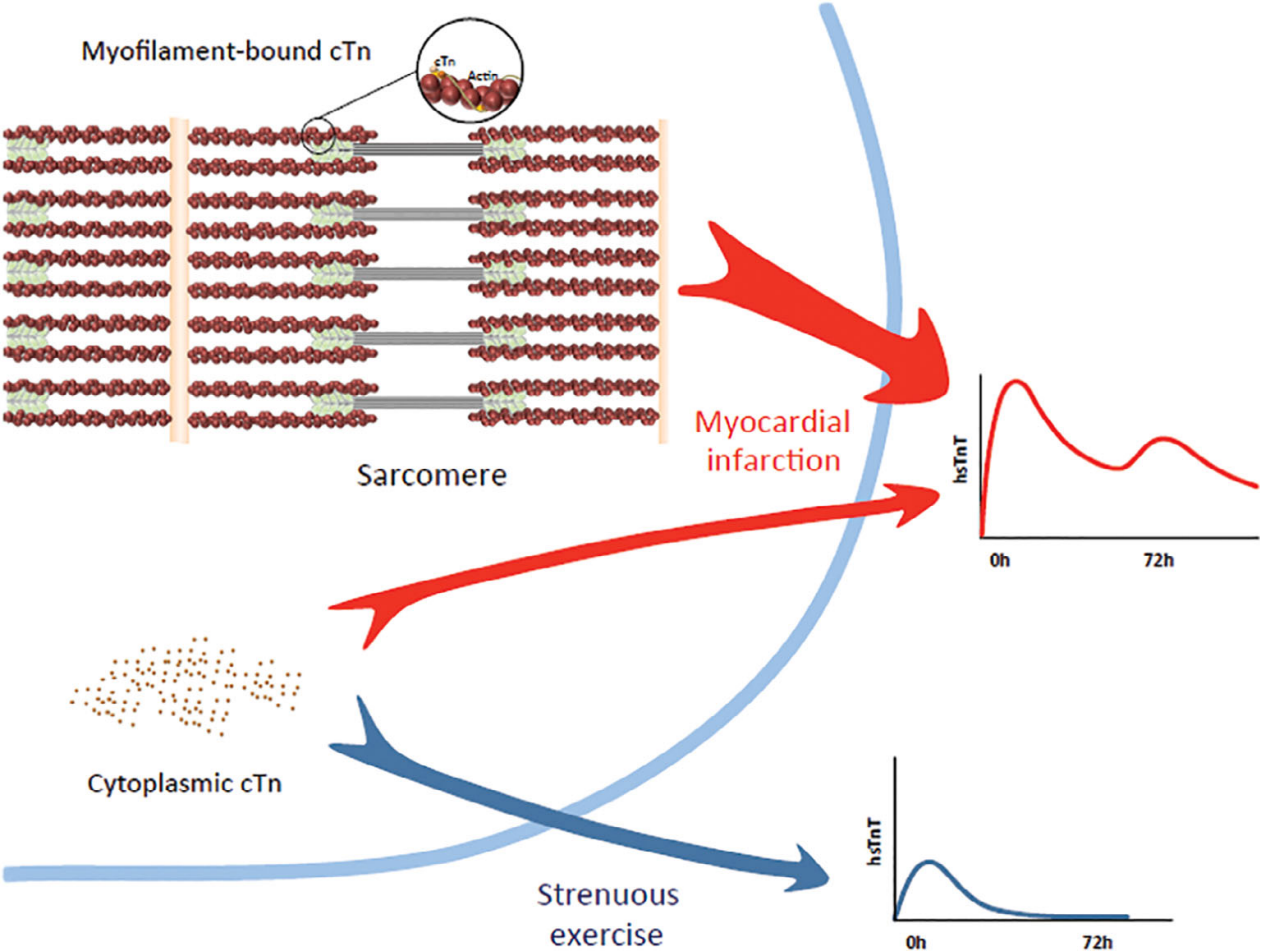
This shows the schematics of the troponin release after myocardial infarction (MI) vs endurance exercises. Prolonged exercise can increase cardiomyocyte membrane permeability and secretion of cytoplasmic‐free cardiac troponin. On the contrary, troponin elevation in MI is due to cardiomyocyte necrosis and release of myofilament‐bound troponin^60^

Consistent with both cytosolic and structural distribution of cTn, the appearance in blood following injury, exhibits a biphasic release pattern.^55^ The kinetics of postexercise Tn release, with an early peak and quick normalization, is quite different from the elevation pattern observed in ACSs,^56^ or even ablation,^57^ indicating that exercise‐induced cTn elevation may arise from the cytosol and not from the thin filaments in the contractile apparatus^58^, ^59^, ^60^(Figure 1).

Another issue to be considered when interpreting cTn levels, is that the antibodies comprising Tn immunoassays may be susceptible to cross reactions induced by immunologic factors or heterophilic antibodies, creating a source of false positive results. Such reactions have been described in relation to the presence of rheumatoid factor,^61^ or diseases like myasthenia.^62^ Various hypotheses have been proposed for the pathophysiologic mechanisms underlying cTn elevation post exercise, including increased membrane permeability, normal turnover of myocardial cells, cTn degradation products cellular release, membranous blebs, myocyte apoptosis/necrosis resulting in genuine cardiac injury, skeletal muscle origin of cTn. The exact etiology remains still speculative due to limited empirical data,^63^, ^64^ but the most common theories are briefly discussed below.

### 4.2 Increased membrane permeability

Starnberg et al^58^ performed in vitro studies from human cardiac tissue and found that transient cytosolic leakage of cytosolic cTn might be facilitated by increases in myocardial sarcolemmal permeability, possibly propagated, among others, by free radical mediated injury.^64^ Free radical generation can be induced by mechanical stress on the cardiomyocytes, increased body temperature, or prolonged acidosis.^65^ Whenever the capacity of the lymphatics to remove macromolecules is exceeded, cTn becomes detectable in the peripheral circulation. This passive diffusion of cTn from the intracellular to extracellular compartment, which has also been described in skeletal muscle exposed to exercise,^66^ is compatible with the release kinetics of cTn observed in this setting; nevertheless, no specialized biological process (eg, exocytosis) has been experimentally proved to serve this scope.^67^

### 4.3 Cardiac injury

Myocardial injury, differentiated by MI and irreversible damage, may be acute, as evidenced by a newly detected dynamic rising and/or falling pattern of cTn, or chronic, in the case of persistently elevated cTn levels.^3^ Exercise provoked mechanical stimuli might induce transient disruptions of the myocardial plasma membrane, termed “cell wounds,” also stimulated by integrins.^68^ This process does not result in frank myocardial injury or cell necrosis, because cardiac regenerative capacity (1% per year in adults over 25 year, 0.3% at the age of 75) would not be sufficient to maintain contractile capacity and compensate the amount of cumulative injury indicated by cTn values observed in repeated sport events.^69^ However, multiple bouts of mechanical stress forces may elicit stretching of the myocytes, stimulated also by inflammatory pathways, as evidenced by interleukin and neutrophil count increase in response to exercise.^70^ These repeated episodes with cTn elevation, followed by immediate restoration of normality may represent a physiological repairing, or remodeling process. In this context, the fibrotic lesions^14^ and calcified, yet stable, atheromatic plaques^13^, ^71^ observed to a greater extent in lifelong endurance athletes, may be the expression of these pathways in the myocardium and arterial wall, respectively. This provides a plausible explanation of equivocal laboratory and imaging results, which, despite initial reports,^72^ do not convincingly connect extreme exercise to adverse cardiovascular events, probably because this benign plaque composition does not often cause plaque rupture and ACS.^73^ In accordance with this, in the study by DeFina et al,^74^ conducted on 21 758 men, higher levels of physical activity were generally associated with more prevalent coronary artery calcification (CAC) (≥100 Agatston Units, AU). Nevertheless, in the group with CAC score of at least 100 AU, physical activity of at least 3000 MET‐min/week was not associated with an increased all‐cause or cardiovascular disease mortality risk compared to less than 1500 MET‐min/week after a decade of follow‐up.

### 4.4 Musculoskeletal etiology

Even though the cardiospecificity of the latest generations of cTnT assays has been validated against skeletal muscle damage,^5^ recent evidence suggests that skeletal muscle injury might cause elevations at least of hs‐cTnT. Numerous papers have appeared describing frequent elevations of hs‐cTnT (usually without concomitant cTnI elevation) in myopathies in the absence of any cardiovascular injury, the latter being excluded after thorough cardiac investigations, including CMR.^75^ It should also be noted that troponin elevations may occur in up to 11% to 30% of cases during rhabdomyolysis, without any signs of cardiac involvement.^76^ Skeletal muscle expression of cTn has been supported by Western blot studies on skeletal muscle biopsies and has been attributed either to cross reaction with troponin isoforms, or to re‐expression of cardiac isoforms in diseased skeletal muscle,^75^ although this has been debated by others.^77^ Lippi et al,^36^ conducting a study based on a model of eccentric exercise, a type of muscular activity sufficient to evoke skeletal, but not myocardial muscle injury, concluded that skeletal muscular injury might affect the concentration of hs‐TnT for up to 13%. Previous other reports have also provided evidence correlating cTn to skeletal muscle, rather than to cardiac parameters (Table 3).^78^ Finally, even if skeletal muscle etiology cannot provide a comprehensive answer, contribution, or interference from skeletal muscle damage should not be overlooked. Numerous studies have demonstrated cTnI elevations post exercise as well; nevertheless, cTnT and cTnI should not be considered interchangeable and this concept is adopted in the recent MI definition.^3^

**Table 3:** In the study by Weippert et al,^78^ a significant association was shown between cTnT release and the metabolic state of the working muscles, that is, exercise “dose,” but not with echocardiographic parameters. Furthermore, Pearson correlation analysis presented on figure 3 (unpublished data, personal communication) reveals strong positive correlation between hs cTnT and total CK/CK‐MB/%CK‐MB

	cTnT post + 1		cTnT post + 4
Total CK post + 1	0.407	Total CK post + 4	0.509
CK‐MB post + 1	−0.172	CK‐MB post + 4	0.155
%CK‐MB post + 1	−0.315	%CK‐MB post + 4	−0.448

Abbreviations: CK‐MB, myocardial band isoform of creatine kinase; cTnT, cardiac troponin T.

## 5 PROGNOSTIC SIGNIFICANCE AND CLINICAL IMPLICATIONS OF EXERCISE‐INDUCED CTN ELEVATION

It has been consistently demonstrated that resting cTn confers an increased risk of adverse cardiovascular outcomes even in the absence of ACS^79^ and can be a prognosticator in various disease states,^17^ or even in healthy individuals.^18^ Although a large spectrum of observations has investigated the relationship between exercise and cTn release, there are only limited data regarding outcomes. Transient exercise‐induced cTn elevation has been generally considered benign, based on the high frequency it occurs in apparently healthy asymptomatic individuals, the lack of correlation with functional (imaging) evidence, and the obvious health benefits provided by endurance exercise. Siegel et al^19^ in a previous study employing first‐generation assays, followed nine runners with a postmarathon cTn increase and showed that all remained asymptomatic for cardiac disease 1 year later. In a study by Möhlenkamp et al^20^ marathon runners with late gadolinium enhancement had higher postrace hsTnI values compared to those without. However, after a 6‐year follow‐up period, coronary event rates were associated with CAC and myocardial fibrosis, but not with increases in hsTnI. Nevertheless, it must be noted that the relatively small number of participants (74) and events recorded (6) might have limited the reliability of this report. In contrast with these evidence, a recent study conducted by Aengevaeren et al^21^ in 725 long‐distance walkers (61 years on average), found that troponin I elevations above the 99th percentile after an ultra‐endurance walking event, was associated with higher mortality and cardiovascular events after 43 months of follow‐up, although this population with relatively increased cardiovascular risk factor burden, may not be quite representative or comparable with most relevant studies of athletes. Therefore, recent reports have seriously disputed the position regarding benign prognosis, indicating that exercise‐induced cTnT elevations may provide incremental prognostic information beyond that of baseline values, not only contributing in unmasking subclinical cardiac pathology,^22^ but potentially serving as an early marker of future cardiovascular events as well.^21^

The potential utility of adding cTn testing in cardiac stress studies, aiming at improving diagnostic accuracy, have so far proved inconsistent^80^; however, under the light of latest reports suggesting new sampling options and prognostic information, this might require reconsideration.

**Figure 2:**
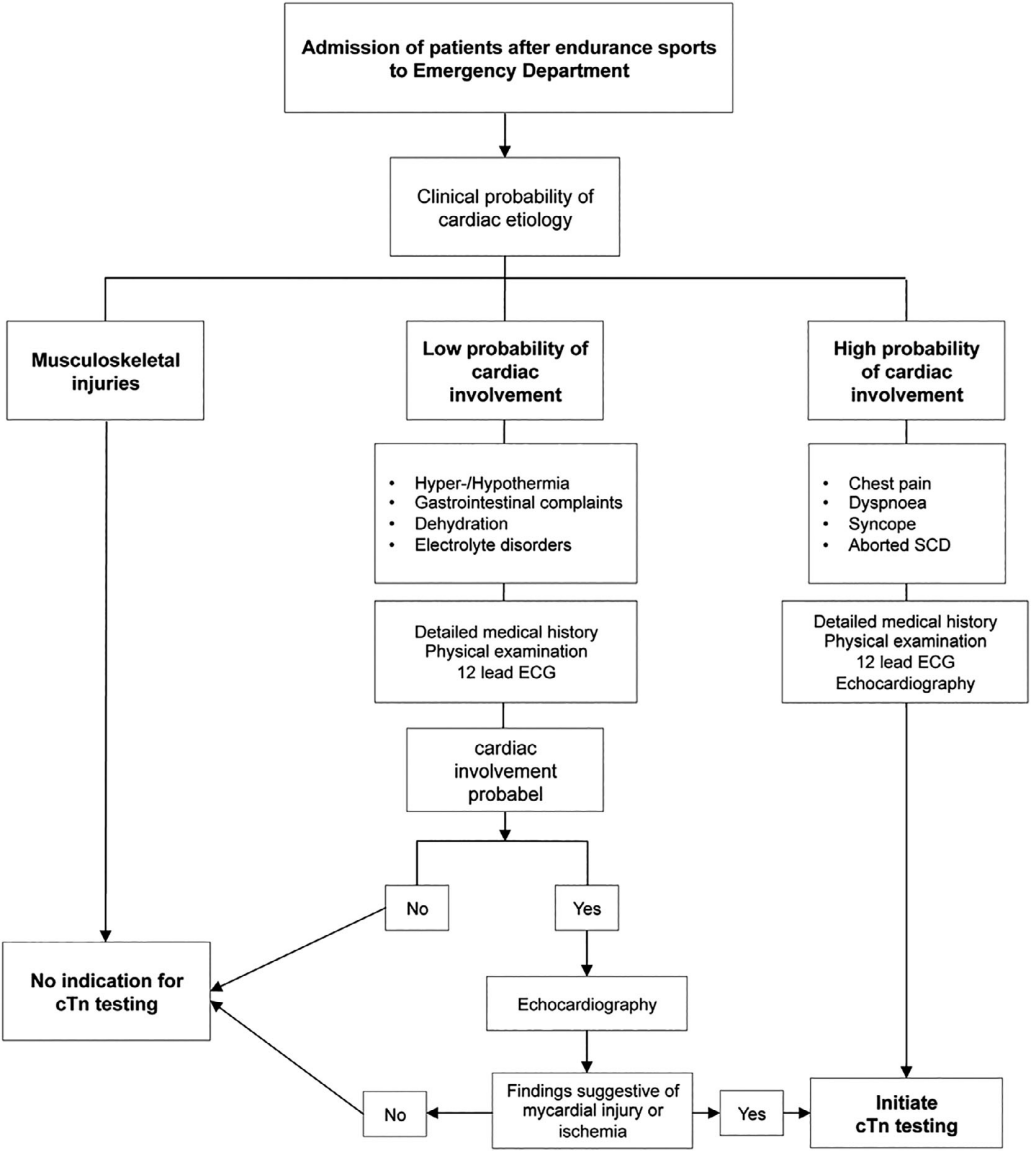
Algorithm outlining proposed management of patients with suspected ACS after prolonged exercise algorithm for the initiation of cardiac troponin (cTn) testing in patients after prolonged exercise, proposed as an adjunct to the standard clinical guidelines for acute coronary syndrome (ACS). ECG, electrocardiogram^60^

Routine cTn testing is not indicated for the evaluation of individuals who present for medical attention after participation in athletic events, unless there are specific clinical signs, symptoms, or data suggesting cardiac involvement.^81^ In cases of suspicious symptoms, a careful workup (Figure 2)^63^ including serial measurements of cTn is recommended. Clinicians should be aware that remarkable cTn elevations might occur routinely after prolonged or strenuous endurance exercise in healthy individuals and in the absence of other signs, symptoms, or data, should avoid extensive but unnecessary investigations. In selected cases with low cardiac risk but unexpectedly high cTnT, additional cTnI or muscle damage markers measurement might be useful. However, whenever serious cTn elevation is followed by obvious skeletal muscle injury, but cardiac involvement cannot be easily excluded (eg, atypical symptoms, high risk factor burden) computed tomography coronary angiography (CTCA), if available, may represent a fast, safe and cost effective “rule out” strategy.^82^

CTCA is favorably incorporated into a triage strategy aiming at improving the diagnostic efficiency in the emergency department, particularly in low‐ to intermediate‐risk patients.^82^ Furthermore, latest data suggest that CTCA may provide information regarding plaque morphology and composition, not only offering insights to pathophysiological mechanisms, but enhancing cardiac risk prediction as well.^83^

## 6 FUTURE DIRECTIONS

Etiological studies in human and animal models may well be important avenues for future study. The determination of adequate cardiac and muscle markers pre and post a strenuous sport event, coupled with multiple imaging could provide further insights into this topic. The relevance of elevated cTn before and after clinical stress tests in order to further stratify patients, might be reconsidered. Finally, large prospective studies are needed to explore whether cTn elevations after physical stress actually confer an incremental risk for future cardiac events independent of conventional risk markers in a wide range of active populations.

## 7 CONCLUSIONS

Although the etiology remains obscure, numerous reports have demonstrated that cTn may be elevated in most subjects following various types of physical activity, ranging from walking to ultra‐endurance sports sessions, in a wide range of populations. It has been suggested that cTn release is correlated with transient cardiac injury induced by strenuous exercise; however, the vast majority of studies do not support and association with changes in cardiac function and/or permanent damage/dysfunction. Consequently, exercise‐induced cTn elevation has been considered a benign phenomenon although this position has been challenged by latest reports. cTn is regarded as highly cardiac specific; nevertheless, it has been recently shown that cTnT increases may be related to skeletal muscle damage. Awareness that cTn elevations are commonly seen after exercise may contribute in avoiding unnecessary procedures in patients admitted to emergency care.
